# A Conservative Weight Loss Intervention Relieves Bowel Symptoms in Morbidly Obese Subjects with Irritable Bowel Syndrome: A Prospective Cohort Study

**DOI:** 10.1155/2018/3732753

**Published:** 2018-03-01

**Authors:** Martin Aasbrenn, Stian Lydersen, Per G. Farup

**Affiliations:** ^1^Department of Surgery, Innlandet Hospital Trust, Gjøvik, Norway; ^2^Unit for Applied Clinical Research, Department of Clinical and Molecular Medicine, Faculty of Medicine and Health Sciences, Norwegian University of Science and Technology, Trondheim, Norway; ^3^Regional Centre for Child and Youth Mental Health and Child Welfare, Faculty of Medicine and Health Sciences, Norwegian University of Science and Technology, Trondheim, Norway; ^4^Department of Research, Innlandet Hospital Trust, Brumunddal, Norway

## Abstract

**Background:**

Irritable bowel syndrome (IBS) is common in subjects with morbid obesity; the effect of weight loss programs on bowel symptoms is largely unknown.

**Methods:**

This prospective cohort study explored bowel symptoms, health scores, and biomarkers in subjects with morbid obesity during a six-month-long conservative weight loss intervention. Bowel symptoms were assessed with IBS-severity scoring system (IBS-SSS) and Gastrointestinal Symptom Rating Scale-IBS. Changes in all variables and associations between the changes in bowel symptoms and the other variables were analysed.

**Results:**

Eighty-eight subjects (81% females) were included. Body mass index was reduced from 42.0 (3.6) to 38.7 (3.5) (*p* < 0.001). IBS-SSS was reduced from 116 (104) to 81 (84) (*p*=0.001). In all, 19 out of 25 variables improved significantly. In subjects with and without IBS at inclusion, the improvement in IBS-SSS was 88 (95% CI 55 to 121) and 10 (95% CI −9 to 29), respectively. Improved bowel symptoms were associated with improved subjective well-being, sense of humour, and vitamin D and negatively associated with reduced body mass index.

**Conclusion:**

Body mass index and health scores improved during a conservative weight loss intervention. Subjects with IBS before the intervention had a clinically significant improvement in bowel symptoms.

## 1. Introduction

Several gastrointestinal disorders have been associated with high body mass index (BMI) in population-based studies, and both upper gastrointestinal disorders and bowel disorders are considered as complications of obesity [1–3]. Knowledge about the effect of weight loss programs on functional bowel disorders is limited [4, 5]. Irritable bowel syndrome (IBS) is a functional bowel disorder that is present in about 11% of the global population and even more common in some groups of patients with morbid obesity (MO) [6, 7]. The main symptom of IBS is recurrent abdominal pain or discomfort related to defecation [8]. IBS has a high impact on quality of life and ability to work and is associated with many comorbidities, including pain syndromes and psychiatric disorders [9].

The aetiology of IBS is an interplay between central psychological factors and peripheral intestinal factors. Some pathophysiological factors have been reported both in patients with IBS and in patients with MO: psychological distress, dietary factors, an altered gut microbiome, and low-grade inflammation [9–17]. Associations between the cytokines secreted by adipose tissue and some of the comorbidities of obesity are plausible, and systemic low-grade inflammation might be a cause of IBS in subjects with MO [18].

Dietary interventions are parts of the treatment of both IBS and MO. A reduced intake of fermentable oligo-, di-, and monosaccharides and polyols (FODMAPs) will often relieve IBS symptoms, and a variety of low-energy diets can lead to weight reduction in subjects with obesity [19, 20].

The primary aim of this study was to compare bowel symptoms and IBS before and after a conservative weight loss intervention. Secondary aims were to study changes in several health scores and biomarkers during the intervention and to explore the associations between the changes in the bowel symptoms and the other variables.

## 2. Materials and Methods

### 2.1. Study Design and Setting

Adults referred to an outpatient obesity clinic in South Eastern Norway from December 2012 to September 2014 were invited to participate in this prospective cohort study. Information was collected at the visits before and after a six-month-long weight loss intervention period. The visits included anthropometric evaluations and retrieval of blood samples. The subjects reported symptoms, demographics, and comorbidity on a paper-based case report form. A physical examination was performed. Additional diagnostic procedures including endoscopic examinations were done at the discretion of the attending physician.

### 2.2. Participants

**Figure 1:**
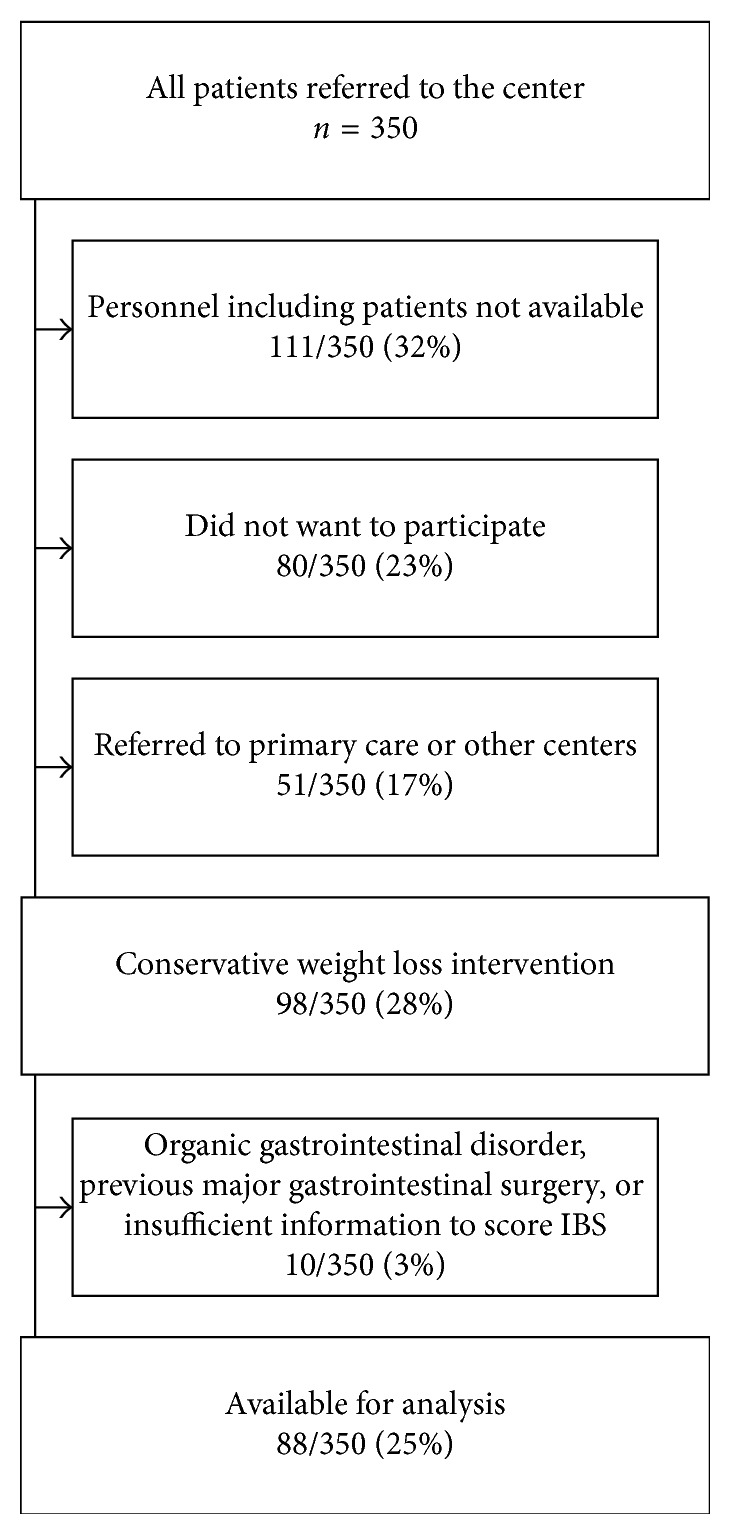
Inclusion of subjects.

The inclusion criteria were age 18–65 years and morbid obesity, defined as either BMI > 40 kg/m^2^ or BMI > 35 kg/m^2^ with complications (diabetes mellitus, hypertension, sleep apnoea, respiratory failure, or musculoskeletal pain related to movement) [21]. Exclusion criteria were organic gastrointestinal disorders; major psychiatric disorders; serious somatic disorders not related to obesity, alcohol, or drug addiction; previous obesity surgery; and other major abdominal surgery. Subjects with insufficient information to assess IBS were also excluded. The inclusion of subjects is depicted in Figure 1.

### 2.3. Intervention

The weight loss intervention lasted for approximately six months. First, the subjects had three separate one-hour-long individual consultations with a nurse, a dietician, and a physician who gave advice on lifestyle and diet. The advice was personalised based on the subjects' exercise habits and preferences, food preferences, and former diet. The time intervals between the appointments were individualised to give the subjects time to implement the changes. In the middle of the six-month intervention period, the subjects were enrolled in groups of eighteen to twenty who had weekly four-hour meetings for seven consecutive weeks. These meetings consisted of group counselling, a lunch together, and lectures by dieticians, physicians, nurses, and a psychologist about awareness and habits.

The lifestyle advice was focused on an increase in enjoyable physical activity, usually hiking in nature together with friends, biking, swimming, or cross-country skiing. The dietary advice was based on the reduction of total energy intake, use of less energy dense food, more fiber and protein, less sugar and fat, and more food rich in micronutrients. The subjects were recommended 4–6 meals per day with 2–4 hour intervals between meals. The last three weeks consisted of a strict “crisp bread diet” with an energy intake of 3765 kJ/day. In this period, the daily food intake was 4.5 dl of low-fat milk, 144 grams of rye-based crisp bread with low-fat, high-protein topping (low-fat cheese, meat, or fish), a small dinner dish (meat or fish), and free amounts of water, beverages without calories, and vegetables (all vegetables were accepted except sweet corn, olives, and avocados). The subjects were allowed to replace the crisp bread diet with meal replacement powder as long as the maximum energy intake remained below 3765 kJ/day. They were informed that acceptance to the public, free-of-charge bariatric surgery program partly depended on adherence to the conservative weight loss program [22].

### 2.4. Variables

#### 2.4.1. Anthropometrics

Height and weight were registered to calculate BMI (kg/m^2^).

#### 2.4.2. Demographics

Five demographic variables were registered: age (years), gender (male/female), smoking habits (daily smoking/not daily smoking), employed (working/not working), and cohabitant status (living with partner/not living with partner).

#### 2.4.3. Diseases

Nine present or previous diseases were registered (yes/no): hypertension, diabetes mellitus, myocardial infarction, stroke, fibromyalgia, psychiatric disorders, allergic rhinitis, chronic obstructive pulmonary disorder, and hypothyroidism.

#### 2.4.4. Health Scores

Thirteen health scores were assessed:

1. *Irritable bowel syndrome-severity scoring system (IBS-SSS)*: this has been developed for the use in clinical and research settings. The score ranges from 0 to 500. Mild, moderate, and severe degrees of IBS have been defined by scores of 75 to 175, 175 to 300, and >300, respectively. Subjects with IBS and a score below 75 are considered to be in remission, and a change of 50 has been judged as clinically significant [23, 24].
2. *Gastrointestinal Symptom Rating Scale modified for use in patients with IBS (GSRS-IBS)*: this scale is an IBS-specific version of the Gastrointestinal Symptom Rating Scale. The score ranges from 1 to 7; high values indicate more discomfort. GSRS-IBS has five subscales: pain, bloating, constipation, diarrhoea, and satiety. The response scale is as follows: (1) no discomfort at all; (2) minor discomfort; (3) mild discomfort; (4) moderate discomfort; (5) moderately severe discomfort; (6) severe discomfort; and (7) very severe discomfort [25].
3. *Rome III criteria*: these criteria were used to diagnose functional bowel disorders [8]. IBS and the IBS subtypes, functional bloating, functional constipation, and functional diarrhoea were noted as present or absent.
4. *Hopkins Symptom Checklist (HSCL-10)*: this is a ten-item questionnaire that measures psychological distress/mental health. The score ranges from 1 to 4. High levels indicate high levels of psychological distress; 1.85 is a cutoff point for normality [26].
5. *WHO-5 Well-Being Index (WHO-5)*: this is a five-item questionnaire that measures subjective well-being. It is a reliable measure of emotional functioning and a screening tool for depression. The index ranges from 0 to 100; high scores indicate better well-being. A score of 50 or below indicates low mood and a score of 28 or less indicates likely depression [27].
6. *Rosenberg's Self-Esteem Scale*: this is a ten-item questionnaire that measures general self-esteem. The scale ranges from 0 to 30; high scores indicate a high self-esteem [28].
7. *Fatigue Severity Scale (FSS)*: this is a nine-item questionnaire that measures fatigue (a sense of physical tiredness and lack of energy, distinct from sadness or weakness). The scale ranges from 1 to 7; high scores indicate a high level of fatigue. A threshold of 5 has been used to define high fatigue [29].
8. *Epworth Sleepiness Scale*: this scale is an eight-item questionnaire that measures general daytime sleepiness. The scale ranges from 0 to 24; high scores indicate higher sleepiness [30].
9. *Sense of Humour Questionnaire (SHQ-6)*: this is a six-item questionnaire that measures the sense of humour. The score ranges from 1 to 4; high scores indicate high sense of humour [31].
10. *Suter's Questionnaire*: this is a questionnaire that measures food tolerance. The food tolerance score ranges from 1 to 27; high scores indicate good food tolerance [32].
11. *Number of meals*: this was assessed with Suter's Questionnaire; both main meals and smaller meals were included in the count [32].
12. *Musculoskeletal pain score*: this is a six-item questionnaire that measures the degree of musculoskeletal pain. It ranges from 0 to 12; high scores indicate more pain.
13. *Physical activity score*: this is a two-item questionnaire. The first question asks about easy activity (not sweaty/breathless), and the second question asks about strenuous activity (sweaty/breathless). There are four alternatives to each question: none, under 1 hour, 1-2 hours, or 3 hours and more per week. For light activity, the four alternatives were scored as 0, 1, 2, and 3, respectively. For strenuous activity, the four answers were scored as 0, 3, 4, and 5, respectively. A score ranging from 0 to 8 was created by adding the scores for easy and strenuous physical activity, and a high score indicates high physical activity.

#### 2.4.5. Blood Tests

Low-density lipoprotein, high-density lipoprotein, cholesterol, C-reactive protein (CRP), thyroid-stimulating hormone, thyroxin, HbA1c, vitamin B_12_, vitamin B_1_, vitamin B_6_, and vitamin D were analysed.

#### 2.4.6. Dietary Intake

In a subset of the patients, the intake of energy and some selected nutrients was registered at inclusion with a semiquantitative food frequency questionnaire designed and validated for the Norwegian population [33].

### 2.5. Statistical Analysis

Data have been presented as mean (standard deviation), median (range), and proportion (percentage) according to the type and distribution of data. The changes in the prevalence rates have been presented with a Newcombe 95% CI [34]. McNemar's test, Wilcoxon signed-rank test, or paired *t*-test was used depending on the type of data and normality. The distributions of IBS categories before and after the intervention were compared with Stuart-Maxwell test for marginal homogeneity for nominal categories [34] and computed in Stata Statistical Software: Release 14 (StataCorp LP, College Station, TX). Two-sided *p* values < 0.05 were judged to indicate statistical significance. When changes in a variable were significantly different in subjects with and without IBS (evaluated with Mann-Whitney *U* test), results were also presented stratified by IBS status.

Linear regression analyses were used for the study of the associations between improvements in IBS-SSS, GSRS-IBS, and BMI (dependent variables) and the improvement in one-by-one of the other variables (independent variables) with sex and age as covariates. Lower thyroid-stimulating hormone, higher thyroxine, and more meals/day were defined as improvements. The results of the linear regression analyses have been presented as B values with 95% confidence intervals, partial correlations (pc), and *p* values. To adjust for multiple testing, Benjamini-Hochberg false discovery rate adjusted *q* values were calculated in *R* [35]. Where not indicated, data analysis was performed with IBM SPSS Statistics for Windows, Version 21.0 (IBM Corp., Armonk, NY).

### 2.6. Ethical Approval

The study was approved by the Regional Committees for Medical and Health Research Ethics South East Norway, reference 2012/966, and conducted in accordance with the Declaration of Helsinki. Written informed consent was obtained from all the subjects included in the study.

## 3. Results

Eighty-eight subjects (71 (81%) females) with a mean age of 44 (SD 8) years were included (Table 1). The prevalence of IBS was 24/88 (27.3%) before and 17/88 (19.3%) after the intervention (Table 2). The change in prevalence was 8.0% (95% CI −18.2% to 2.4%, *p*=0.126). GSRS-IBS showed a reduction in overall symptoms, bloating, diarrhoea, and satiety and an increase in constipation (Table 3). The distribution of IBS subtypes before and after the intervention did not differ significantly (*p*=0.36). In subjects with and without IBS at the first visit, the improvement in IBS-SSS was 88 (95% CI 55 to 121) and 10 (95% CI −9 to 29), respectively (Table 4).

BMI was reduced from 42.0 (SD 3.6) to 38.7 (SD 3.5) kg/m^2^. The change in BMI was 3.3 kg/m^2^ (95% CI 3.0 kg/m^2^ to 3.6 kg/m^2^, *p* < 0.001). Psychological distress, subjective well-being, self-esteem, fatigue, sleepiness, and musculoskeletal pain improved. In blood, CRP, cholesterol, and low-density lipoprotein decreased and the levels of vitamin B_6_, B_12_, and D increased (Table 3). The recommended energy intake at the end of the intervention was less than half of the self-reported energy intake before the intervention, while the intake of bread and milk largely was unchanged (Table 5).

The improvement in IBS-SSS was associated with an improved sense of humour (pc = 0.30; *p*=0.012) and was negatively associated with improvement in BMI (pc = −0.29; *p*=0.012). The improvement in GSRS-IBS was associated with improvement in emotional well-being (pc = 0.23; *p*=0.038) and vitamin D in plasma (pc = 0.29; *p*=0.010). Neither the changes in IBS-SSS nor GSRS-IBS were significantly associated with the changes in the other health scores or blood tests. None of the associations between improvements remained significant after adjustment for multiple testing (Table 6).

## 4. Discussion

### 4.1. Effects of the Intervention

Nineteen out of 25 variables improved significantly during the weight loss intervention, including 9 out of 13 health scores. IBS-SSS and GSRS-IBS, the health scores that measure bowel symptoms, were two of the variables that improved. Subscales of GSRS-IBS showed that bloating, satiety, and diarrhoea were relieved, while constipation worsened. The prevalence of IBS was reduced, but not significantly. It was the subjects with IBS at inclusion who had the most marked improvement in bowel symptoms. In this group, the whole confidence interval for the IBS-SSS improvement was above the limit for a clinically significant effect. Subjects without IBS at inclusion did not have significant changes in IBS-SSS and GSRS-IBS.

BMI was reduced by 3.3 kg/m^2^, achieved by a conservative weight loss intervention based on lifestyle changes with dietary modifications and increased physical activity. The frequent follow-up in groups, the clear expectations from the health care personnel, and the information that good compliance was a prerequisite for bariatric surgery in the public health system after the intervention were crucial for the satisfactory results.

According to the recommendations in the program, the number of meals per day increased. Lower energy intake and a healthier choice of macronutrients (less saturated fats and sugar) were probable causes of the reductions in serum cholesterol and low-density lipoprotein [36]. Healthier food could explain parts of the increase in serum vitamin levels. Vitamin D levels might also have increased due to sun exposure related to outdoor activities and reduced fat mass [37]. Reduced secretion of adipokines from the visceral adipose tissue and some increase in physical activity account for the expected fall in CRP values [38]. Psychological effects related to the successful weight loss, the social support, and some increase in physical activity were probably important causes of the improved psychological distress, subjective well-being, self-esteem, fatigue, sleepiness, and musculoskeletal pain.

### 4.2. Variables Associated with Bowel Symptom Improvement

The second part of the analysis aimed to identify associations between changes in bowel symptoms and changes in other variables. The social support and positive psychosocial environment achieved during the intervention is a likely explanation for the association between improved bowel symptoms and improvement in subjective well-being and sense of humour. Psychosocial factors are of importance for the symptom load in subjects with IBS [9].

The negative association between the improvements in IBS-SSS and BMI was unexpected. The diet in this specific conservative weight loss program was based on FODMAP-rich food (rye-based crisp bread, milk, and vegetables as cauliflower and peas). Even though energy intake was heavily reduced, absolute intake of the main FODMAP sources in a Norwegian diet was mainly unchanged. The relative amount of FODMAPs in the food was therefore increased, especially in subjects with strict adherence to recommendations, which might be an explanation of the negative association between improvement in bowel symptoms and BMI.

Reduction of GSRS-IBS was associated with an increase in serum vitamin D, and the improvements in bowel symptoms could be related to other changes in diet, such as more regular eating of high-quality food rich in micronutrients. Changes in CRP were not associated with changes in bowel symptoms. Seen together with the observation that weight loss itself was negatively correlated with bowel symptom improvement, our data do not support the hypothesis that systemic low-grade inflammation from adipose tissue is the main mediator that leads to IBS in the obese [18]. However, the observed improvement might be due to interplay between several peripheral factors. For instance, the gut microbiota is associated with both IBS and MO [16, 17], and the interaction between peripheral gut factors as food, microbiota, and local gut inflammation could plausibly change gut symptoms.

### 4.3. Clinical Implications

Improvement in BMI and many health scores and biomarkers was observed, and the study adds to the evidence that a conservative weight loss program can lead to better health in the short term [22]. Comorbidity should be taken into account when choosing between treatment alternatives for subjects with MO [19]. This study indicates that when MO and IBS coexist, nonsurgical treatment should be considered, as surgery-induced weight loss probably leads to increased abdominal pain and bowel symptoms [5, 39].

The degree of improvement in IBS-SSS from this lifestyle intervention in subjects with IBS and MO was comparable to the improvement seen after IBS-specific diets in normal weight subjects [24]. It is probable that the effect of conservative weight loss intervention on bowel symptoms can be made even larger if FODMAPs are taken into account.

### 4.4. Strengths and Limitations

The weight loss program has been developed and used with favorable experience for a long time in several Norwegian clinics for morbid obesity [22]. The program functioned well, and the loss to follow-up during the six-month period was low. The amount of weight change achieved by the subjects was satisfactory, and the study was large enough to show a statistically and clinically significant improvement in bowel symptoms and many other variables. Validated instruments were used for the measurements of the bowel symptoms and the health-related variables [8, 23, 25–30, 32].

Intake of FODMAPs was not measured in individual participants after the intervention, and registration of upper gastrointestinal disorders was not done. Fat distribution was not measured with either waist/hip ratio or radiological methods, and the compliance with advice (diet and physical activity) given during the intervention was unknown. None of the associations between changes remained significant after adjustment for multiple testing, which limits the strength of the findings. The generalizability of the results to subjects with BMI under 35 is unknown. The Rome III criteria demand symptoms of at least six months duration to diagnose IBS, which limits the diagnosis of IBS in subjects with new symptoms. A longer study period had been preferable.

## 5. Conclusions

BMI was reduced and health improved during a conservative weight loss program for subjects with MO. Subjects with IBS and MO also experienced a clinically significant improvement in IBS symptoms. As bowel symptoms often aggravate after bariatric surgery, conservative treatment should be considered as an alternative in subjects with MO and IBS if medically advisable. Psychosocial changes and possibly a more healthy and regular diet could explain the improvement in bowel symptoms.

## Figures and Tables

**Table 1:** Characteristics of the included subjects.

	Mean (SD) or number (percentage)
Age (years) (*n* = 88)	44 (8)
Male gender	17/88 (19%)
Height (cm) (*n* = 88)	171 (9)
Weight (kg) (*n* = 88)	123 (18)
Body mass index (kg/m^2^) (*n* = 88)	42.0 (3.6)
Daily smoking	18/88 (21%)
Working	68/87 (78%)
Married/cohabitant	65/76 (86%)
Hypertension	29/85 (34%)
Diabetes mellitus	14/86 (16%)
Myocardial infarction	2/86 (2%)
Stroke	3/86 (4%)
Fibromyalgia	20/87 (23%)
Minor psychiatric disorders	18/87 (21%)
Allergic rhinitis	13/86 (15%)
Chronic obstructive pulmonary disorder	2/85 (2%)
Hypothyroidism	13/85 (15%)
Fibromyalgia	20/87 (23%)

**Table 2:** Number (proportion) of subjects with irritable bowel syndrome (IBS) before and after the weight loss intervention.

	IBS after intervention	Without IBS after intervention	Sum
IBS before intervention	10 (11.4%)	14 (15.9%)	24 (27.3%)
Without IBS before intervention	7 (8.0%)	57 (64.7%)	64 (72.7%)
Sum	17 (19.3%)	71 (80.7%)	88 (100%)

Prevalence of IBS before intervention is 27.3% and after intervention is 19.3%. Difference between before and after intervention is 8.0% (Newcombe 95% CI −18.2% to 2.4%). McNemar's asymptotic *p* value is 0.126.

**Table 3:** Subjects' characteristics before and after the weight loss intervention with analyses of the changes.

	Before intervention		After intervention		Change during the intervention	
		*n*		*n*	*p* values	*n*
Body mass index (kg/m^2^)	42.0 (3.6)	88	38.7 (3.5)	87	**<0.001** ^**#**^	87
Weight	123 (18)	88	113 (17)	87	**<0.001** ^**#**^	87
Irritable bowel syndrome (IBS)	24/88 (27%)		17/88 (19%)		0.19^¤^	
Constipation	5/24 (21%)		4/17 (24%)		0.45^&^	
Diarrhoea	8/24 (33%)		7/17 (41%)			
Mixed	11/24 (46%)		6/17 (35%)			
Unsubtyped	0/24 (0%)		0/17 (0%)			
Functional constipation	3/88 (3%)		8/88 (9%)		0.23^¤^	
Functional diarrhoea	3/88 (3%)		3/88 (3%)		1.00^¤^	
Functional bloating	14/87 (16%)		11/87 (13%)		0.66^¤^	
IBS-severity scoring system (IBS-SSS)	116 (104)	84	81 (84)	81	0.001^∗^	77
Gastrointestinal Symptom Rating Scale-irritable bowel syndrome (GSRS-IBS) version	1.8 (0.8)	85	1.6 (0.6)	88	0.006^∗^	85
Pain syndrome	1.9 (1.1)	85	1.7 (0.9)	88	0.18^∗^	85
Bloating syndrome	2.2 (1.3)	85	1.7 (0.9)	88	0.001^∗^	85
Constipation syndrome	1.5 (0.9)	85	1.8 (1.2)	88	0.03^∗^	85
Diarrhoea syndrome	1.8 (0.9)	85	1.5 (0.7)	88	0.02^∗^	85
Satiety	1.6 (1.1)	85	1.2 (0.5)	88	0.001^∗^	85
Hopkins Symptom Checklist (HSCL-10)	1.6 (0.5)	88	1.4 (0.5)	88	**<**0.001^∗^	88
WHO-5 Well-Being Index (WHO-5)	56 (19)	88	65 (14)	88	**<0.001** ^**#**^	88
Rosenberg's Self-Esteem Scale	2.8 (0.5)	87	3.0 (0.5)	86	**0.001** ^**#**^	85
Fatigue Severity Scale (FSS)	37 (15)	87	34 (14)	86	**0.001** ^**#**^	85
Epworth Sleepiness Scale	8 (5)	88	7 (5)	87	**<**0.001^∗^	87
Sense of Humour Questionnaire	3.2 (0.4)	87	3.3 (0.4)	87	0.14^∗^	86
Musculoskeletal pain score	4.5 (3)	87	3.4 (3)	87	**<**0.001^∗^	86
Physical activity score	4.3 (0.2)	88	4.8 (2.2)	88	0.12^∗^	88
Food tolerance (Suter)	24.0 (2.5)	85	24.2 (2.2)	88	0.47^∗^	85
Number of meals/day	4.0 (1.0)	82	4.7 (1.1)	82	**<**0.001^∗^	82
LDL (1.5–4.8 mmol/L)	3.3 (0.9)	87	2.8 (0.8)	84	**<0.001** ^**#**^	84
HDL (F: 1.0–2.7 mmol/L; M: 0.8–2.1 mmol/L)	1.2 (0.3)	87	1.1 (0.3)	85	**<**0.001^∗^	84
Cholesterol (3.3–6.9 mmol/L)	5.1 (1.0)	87	4.3 (0.9)	85	**<0.001** ^**#**^	84
CRP (<5 mg/L)	7 (6)	87	4 (4)	87	**<**0.001^∗^	86
HbA1c (4.0–6.0%)	5.8 (1.1)	87	5.5 (0.8)	85	**<**0.001^∗^	84
TSH (0.27–4.20 mIE/L)	1.9 (1.1)	87	1.6 (1.1)	85	0.002^∗^	84
T_4_ (12–22 pmol/L)	15 (3)	87	17 (7)	85	**<**0.001^∗^	84
Vitamin B_1_ (95–200 nmol/L)	156 (23)	87	163 (30)	85	0.10^∗^	84
Vitamin B_6_ (15–160 nmol/L)	33 (32)	86	62 (44)	85	**<**0.001^∗^	83
Vitamin B_12_ (140–600 pmol/L)	364 (162)	87	433 (231)	85	**<**0.001^∗^	84
Vitamin D (50–150 nmol/L)	55 (21)	87	61 (26)	85	**0.009** ^#^	84

The values are given as mean (SD) or *n* (%). LDL: low-density lipoprotein; HDL: high-density lipoprotein; CRP: C-reactive protein; TSH: thyroid-stimulating hormone; T_4_: thyroxin. Statistical tests with ^¤^McNemar's test, ^&^Stuart-Maxwell test, ^∗^Wilcoxon signed rank test, or ^#^paired *t*-test.

**Table 4:** Subjects' characteristics before and after the conservative weight loss intervention, stratified by IBS status before the intervention.

	Subjects with IBS						Subjects without IBS						
	Before intervention		After intervention		Change during the intervention		Before intervention		After intervention		Change during the intervention		Differences in changes between subjects with and without IBS
		*n*		*n*	*p* values	*n*		*n*		*n*	*p* values	*n*	*p* values
IBS-severity scoring system (IBS-SSS)	218 (89)	24	132 (83)	21	**<**0.001^∗^	21	75 (79)	60	63 (77)	60	0.23^∗^	56	**<0.001** ^**#**^
Gastrointestinal Symptom Rating Scale-irritable bowel syndrome version (GSRS-IBS)	2.6 (0.7)	24	2.0 (0.7)	24	0.001^∗^	24	1.5 (0.6)	61	1.5 (0.6)	64	0.57^∗^	61	**<0.001** ^**#**^
Pain syndrome	3.0 (1.0)	24	2.2 (1.1)	24	0.01^∗^	24	1.4 (0.7)	60	1.5 (0.8)	64	0.29^∗^	60	**0.001** ^**#**^
Bloating syndrome	3.2 (1.3)	24	2.1 (1.0)	24	0.003^∗^	24	1.8 (1.0)	61	1.6 (0.8)	64	0.06^∗^	61	**0.018** ^**#**^
Constipation syndrome	1.9 (1.3)	24	2.3 (1.5)	24	0.32^∗^	24	1.3 (0.6)	61	1.6 (1.1)	64	0.03^∗^	61	0.81^**#**^
Diarrhoea syndrome	2.5 (1.1)	24	1.9 (1.0)	24	0.006^∗^	24	1.5 (0.7)	61	1.4 (0.6)	64	0.46	61	**0.010** ^**#**^
Satiety	2.2 (1.5)	24	1.4 (0.6)	24	0.012^∗^	24	1.4 (0.7)	61	1.2 (0.5)	64	0.03^∗^	61	0.053^#^
Number of meals/day	4.1 (0.9)	23	4.5 (1.1)	22	0.26^∗^	21	3.9 (1.0)	62	4.8 (1.1)	62	**<**0.001^∗^	61	**0.015** ^**#**^
Hopkins Symptom Checklist (HSCL-10)	1.9 (0.6)	24	1.6 (0.6)	24	0.002^∗^	24	1.5 (0.5)	64	1.4 (0.5)	64	**0.006**	64	**0.044** ^**#**^

The values are given as mean (SD) or *n* (%). All variables from Table 3 where the change is significantly different in subjects with and without IBS are included in this table. Statistical tests with ^∗^Wilcoxon signed rank test and ^#^Mann-Whitney *U* test.

**Table 5:** Comparison of intake of energy, bread and milk, before and during the last three weeks of the intervention.

		Measured before the intervention					Recommended during the intervention
	All subjects		Subjects with IBS		Subjects without IBS		
		*n*		*n*		*n*	
Energy (kJ)	10,458 (3890)	68	11,778 (4377)	19	9949 (3601)	49	3765
Intake of milk (ml)	276 (0–1791)	68	304 (5–1791)	19	276 (0–1422)	49	450
Intake of bread (g)	156 (24–338)	68	170 (54–338)	19	165 (24–276)	49	144

Results are given as mean (standard deviation) or median (range) depending on the distribution. The food intake before the intervention was registered with food frequency questionnaires and is presented next to the specific recommendations, given the last three weeks of the intervention.

**Table 6:** Associations between the improvement in bowel symptoms and the improvement in the other variables.

	IBS-severity scoring system (IBS-SSS)				Gastrointestinal Symptom Rating Scale-IBS (GSRS-IBS) version			
	*B* (95% CI)	pc	*p* value	*q* value	*B* (95% CI)	pc	*p* value	*q* value
Male gender	15.1 (−30.8; 61.1)	0.08	0.51		−0.01 (−0.38; 0.35)	0.01	0.95	
Age	1.8 (−0.3; 3.9)	0.19	0.094		0.012 (−0.005; 0.029)	0.15	0.18	
Body mass index	−**15.2 (**−**27.0**; −**3.5)**	−**0.29**	**0.012**	0.13	−0.07 (−0.17; 0.03)	−0.15	0.18	0.48
HSCL-10	23.5 (−26.9; 74.1)	0.11	0.36	0.97	0.28 (−0.11; 0.68)	0.16	0.15	0.48
WHO-5	1.3 (−0.1; 2.6)	0.22	0.059	0.43	**0.011 (−0.001**; **0.021)**	**0.23**	**0.038**	0.42
Self-esteem	42.8 (−13.1; 98.7)	0.18	0.13	0.79	0.12 (−0.31; 0.54)	0.06	0.58	0.79
FSS	0.68 (−1.34; 2.69)	0.08	0.51	0.97	0.003 (−0.013; 0.019)	0.04	0.70	0.79
Sleepiness	−0.26 (−5.46; 4.94)	−0.01	0.92	0.97	0.02 (−0.022; 0.062)	0.10	0.35	0.60
Sense of humour	**74.7 (17.9; 131.4)**	**0.30**	**0.012**	0.13	0.35 (−0.11; 0.81)	0.17	0.14	0.48
Musculoskeletal pain	0.18 (−0.20; 9.55)	0.01	0.97	0.97	0.012 (−0.061; 0.084)	0.04	0.75	0.79
Physical activity	4.09 (−3.15; 11.33)	0.13	0.26	0.73	−0.013 (−0.068; 0.043)	−0.05	0.65	0.79
Number of meals/day	0.05 (−0.07; 0.16)	0.09	0.44	0.97	0.11 (−0.03; 0.01)	0.16	0.16	0.48
Food tolerance	3.02 (5.67; 11.70)	0.08	0.49	0.97	0.04 (−0.03; 0.10)	0.12	0.31	0.60
LDL	8.8 (−24.8; 42.4)	0.06	0.60	0.97	0.10 (−0.17; 0.37)	0.08	0.46	0.72
HDL	−11.0 (−110.6; 88.6)	−0.03	0.83	0.97	−0.75 (−1.54; 0.04)	−0.21	0.061	0.45
Cholesterol	3.0 (−26.4; 32.4)	0.03	0.84	0.97	0.12 (−0.13; 0.36)	0.11	0.35	0.60
CRP	−0.4 (−4.7; 4.0)	−0.02	0.87	0.97	0.003 (−0.033; 0.039)	0.02	0.87	0.87
HbA1c	−8.2 (−40.9; 24.4)	−0.06	0.62	0.97	−0.08 (−0.35; 0.19)	−0.07	0.54	0.79
TSH (decrease)	3.1 (−15.2; 21.4)	0.04	0.74	0.97	0.02 (−0.12; 0.17)	0.04	0.74	0.79
T_4_ (increase)	0.7 (−2.2; 3.6)	0.06	0.63	0.97	0.004 (−0.020; 0.028)	0.04	0.75	0.79
Vitamin B_1_	0.24 (−0.51; 0.99)	0.08	0.53	0.97	0.005 (−0.001; 0.011)	0.17	0.13	0.48
Vitamin B_6_	−0.01 (−0.49; 0.47)	−0.01	0.97	0.97	0.002 (−0.002; 0.006)	0.14	0.23	0.51
Vitamin B_12_	−0.01 (−0.15; 0.14)	−0.01	0.91	0.97	0.0005 (0.0003; 0.0014)	0.14	0.21	0.51
Vitamin D	0.52 (−0.43; 1.46)	0.13	0.28	0.97	**0.010 (**−**0.002**; **0.017)**	**0.29**	**0.010**	0.22

HSCL-10: Hopkins Symptom Checklist 10; WHO-5: World Health Organization 5 Well-Being Index; FSS: Fatigue Severity Scale; LDL: low-density lipoprotein; HDL: high-density lipoprotein; CRP: C-reactive protein; TSH: thyroid-stimulating hormone; T_4_: thyroxin. Linear regression with IBS-SSS and GSRS-IBS as the independent variables, and the other variables one by one as covariates, adjusted for gender and age (gender and age presented unadjusted in the first two rows). *q* values are Benjamini-Hochberg adjusted to preserve false discovery rate for twenty-two hypotheses.
